# Supplementary material for: Causal Relationship between Aging and Anorexia Nervosa: A White-Matter-Microstructure-Mediated Mendelian Randomization Analysis
Source: Biomedicines. 2024 Aug 16;12(8):1874. doi: 10.3390/biomedicines12081874 (PMC11351342; doi:10.3390/biomedicines12081874)
Supplement: Supplementary file 1 [file biomedicines-12-01874-s001.zip › Supplementary Figure/Figure S9/Leaveoneout plot of MR analysis for ubm-b-1842 to AN.pdf]

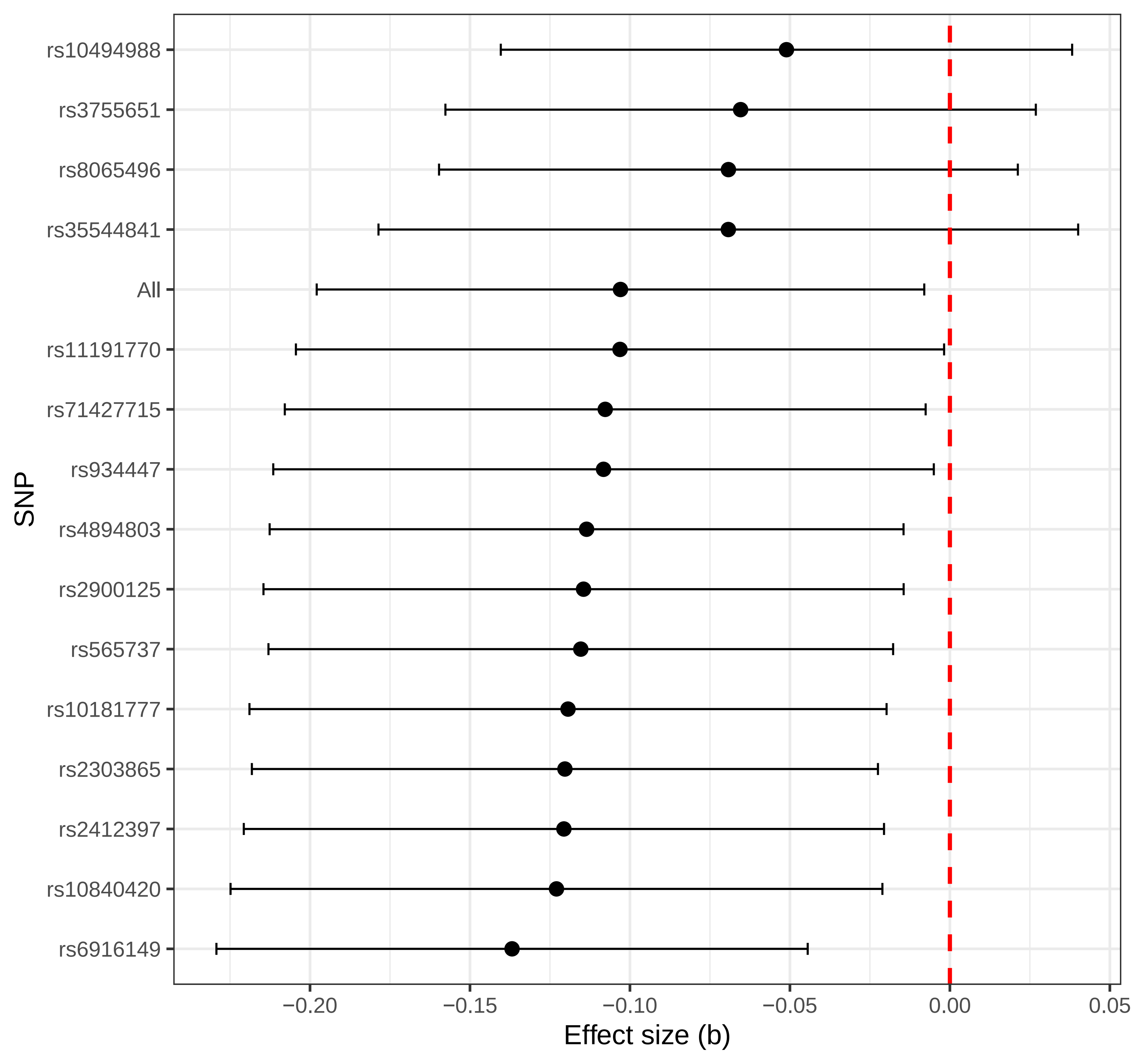
rs10494988
rs3755651
rs8065496
rs35544841
All
rs11191770
rs71427715
rs934447
rs4894803
rs2900125
rs565737
rs10181777
rs2303865
rs2412397
rs10840420
rs6916149
SNP
−0.20
−0.15
−0.10
−0.05
0.00
0.05
Effect size (b)
